# Dynamic effective connectivity in resting state fMRI

**DOI:** 10.1016/j.neuroimage.2017.11.033

**Published:** 2018-10-15

**Authors:** Hae-Jeong Park, Karl J. Friston, Chongwon Pae, Bumhee Park, Adeel Razi

**Affiliations:** a Department of Nuclear Medicine, Radiology and Psychiatry, Yonsei University College of Medicine, Seoul, Republic of Korea; b Center for Systems and Translational Brain Sciences, Institute of Human Complexity and Systems Science, Department of Cognitive Science, Yonsei University, Seoul, Republic of Korea; c BK21 PLUS Project for Medical Science, Yonsei University College of Medicine, Seoul, Republic of Korea; d The Wellcome Trust Centre for Neuroimaging, University College London, London, UK; e Department of Statistics, Hankuk University of Foreign Studies, Yong-In, Republic of Korea; f Department of Electronic Engineering, NED University of Engineering and Technology, Karachi, Pakistan; g Monash Biomedical Imaging and Monash Institute of Cognitive & Clinical Neurosciences, Monash University, Clayton, Australia

## Abstract

Context-sensitive and activity-dependent fluctuations in connectivity underlie functional integration in the brain and have been studied widely in terms of synaptic plasticity, learning and condition-specific (e.g., attentional) modulations of synaptic efficacy. This dynamic aspect of brain connectivity has recently attracted a lot of attention in the resting state fMRI community. To explain dynamic functional connectivity in terms of directed effective connectivity among brain regions, we introduce a novel method to identify dynamic effective connectivity using spectral dynamic causal modelling (spDCM). We used parametric empirical Bayes (PEB) to model fluctuations in directed coupling over consecutive windows of resting state fMRI time series. Hierarchical PEB can model random effects on connectivity parameters at the second (between-window) level given connectivity estimates from the first (within-window) level. In this work, we used a discrete cosine transform basis set or eigenvariates (i.e., expression of principal components) to model fluctuations in effective connectivity over windows. We evaluated the ensuing dynamic effective connectivity in terms of the consistency of baseline connectivity within default mode network (DMN), using the resting state fMRI from Human Connectome Project (HCP). To model group-level baseline and dynamic effective connectivity for DMN, we extended the PEB approach by conducting a multilevel PEB analysis of between-session and between-subject group effects. Model comparison clearly spoke to dynamic fluctuations in effective connectivity – and the dynamic functional connectivity these changes explain. Furthermore, baseline effective connectivity was consistent across independent sessions – and notably more consistent than estimates based upon conventional models. This work illustrates the advantage of hierarchical modelling with spDCM, in characterizing the dynamics of effective connectivity.

## Highlights

• We describe efficient estimation of dynamics in resting state effective connectivity.
• Spectral DCM and PEB are used to model fluctuations in neuronal coupling over time.
• Dynamics in responses are explained in terms of its causes (effective connectivity).
• Baseline and dynamic components of the default mode connectivity are identified.

## Introduction

The human brain exhibits coherent endogenous fluctuations across distributed brain regions in resting-state functional magnetic resonance imaging data (rsfMRI), which are thought to reflect the underlying network architecture (Biswal et al., 1995, Greicius et al., 2003, Lowe et al., 1998, McGuire et al., 1996, Raichle and Snyder, 2007). The temporal coherence among endogenous fluctuations in distributed brain regions – referred to functional connectivity – has attracted an unprecedented interest from the neuroimaging community. Early studies of functional connectivity assumed temporal stationarity, focusing on measures of statistical dependency (e.g., correlation) evaluated over the entire time series. However, a growing number of studies have looked at correlations over shorter time scales to reveal fluctuations in functional connectivity (Allen et al., 2014, Calhoun et al., 2014, Chang and Glover, 2010, Cribben et al., 2012, Handwerker et al., 2012, Hutchison et al., 2013, Monti et al., 2014, Wang et al., 2016). These studies have led to the notion of dynamic functional connectivity that is conventionally estimated using the cross-correlation of rsfMRI. However, functional connectivity – or fluctuations in functional connectivity – do not provide information about the directed casual interactions among brain regions (for review, see Park and Friston, 2013). Furthermore, correlations between haemodynamic measures may not reflect correlations among neuronal activity. These limitations call for a characterization in terms of effective connectivity; namely the causal influence that one neural system exerts over another, either at a synaptic or a population level (Friston, 2011).

This paper describes how to estimate dynamic effective connectivity in the resting state brain, using dynamic causal modelling (DCM). DCM assumes a bilinear model of neural dynamics together with a nonlinear haemodynamic response model of fMRI data (Friston et al., 2003). DCM was initially developed to model task (stimulus)-driven changes in the effective connectivity. To estimate intrinsic (i.e. resting state) effective connectivity from resting state fMRI data, Friston et al. (2014) introduced spectral dynamic causal modelling (spDCM) using the cross-spectra of the blood oxygenation level dependency (BOLD) signals. These cross spectra can be regarded as a more complete measure of functional connectivity because their (inverse) Fourier transform is effectively the cross-correlation function (that includes the conventional (Pearson's) correlation at zero lag). In other words, spectral DCM estimates the effective connectivity that causes or explains functional connectivity. Subsequently, Razi et al. (2015) showed that spDCM reliably estimates intrinsic effective connectivity in the absence of external stimulation. Briefly, spDCM explains the observed cross-spectra using a multivariate autoregressive model (MAR) of the BOLD signals over a period of time. Thus, spDCM can be considered to estimate the effective connectivity that produces the average functional connectivity over the time period examined. However, fluctuations in effective connectivity – over short time periods – during the resting state have not been previously characterized.

In this study, we hypothesized that intrinsic effective connectivity has dynamics that explain dynamic functional connectivity – and which can be modeled with baseline connectivity and a dynamic component that fluctuates about the baseline. We further hypothesized that dynamic effective connectivity could be modeled as a linear combination of orthogonal temporal basis functions. We further expected that when rsfMRI is measured across multiple sessions, either on the same day or different days, the baseline effective connectivity would be stable across different sessions. In other words, the unique aspects of connectivity across different sessions would be the dynamic components.

Under this assumption, we developed a method to estimate baseline and dynamic effective connectivity from rsfMRI using a sliding-window approach, which divides a time-series into a regular number of windows. To estimate fluctuations in effective connectivity over windows, we used the hierarchical framework of parametric empirical Bayes (PEB) (Friston et al., 2015, Friston et al., 2016) to model baseline and dynamic effective connectivity components. In this setting, we first estimate (or invert) a spDCM for each window and then apply PEB to model random (between-window) effects on coupling parameters that are estimated at the first (within-window) level. The second level model is based on a design matrix comprising an orthogonal temporal basis set. Similar approaches have been used previously to estimate the spatiotemporal dynamics of seizure activity based on DCM for EEG; either using Bayesian belief updating (Cooray et al., 2016) or using PEB with tonic and monotonic changes as temporal basis functions at the second level (Papadopoulou et al., 2017). The current study differs from Papadopoulou et al. (2017); not only in its the application to rsfMRI but also in the estimation of connectivity dynamics in the absence of any external inputs or state change markers (such as seizure makers). We further extended the PEB approach to derive group-average intrinsic effective connectivity by conducting additional two-level PEB analysis of the between-session and between-subject group effects. In summary, we applied two level PEB models to identify baseline and dynamic effective connectivity at the session level – and group-averaged baseline connectivity of the default mode network (DMN) using the rsfMRI data from the Human Connectome Project (HCP) database (Van Essen et al., 2012). In doing so, we hope to illustrate the utility of PEB for DCM in quantifying dynamic effective connectivity at rest.

## Materials and methods

### Data and image processing

**Fig. 1:**
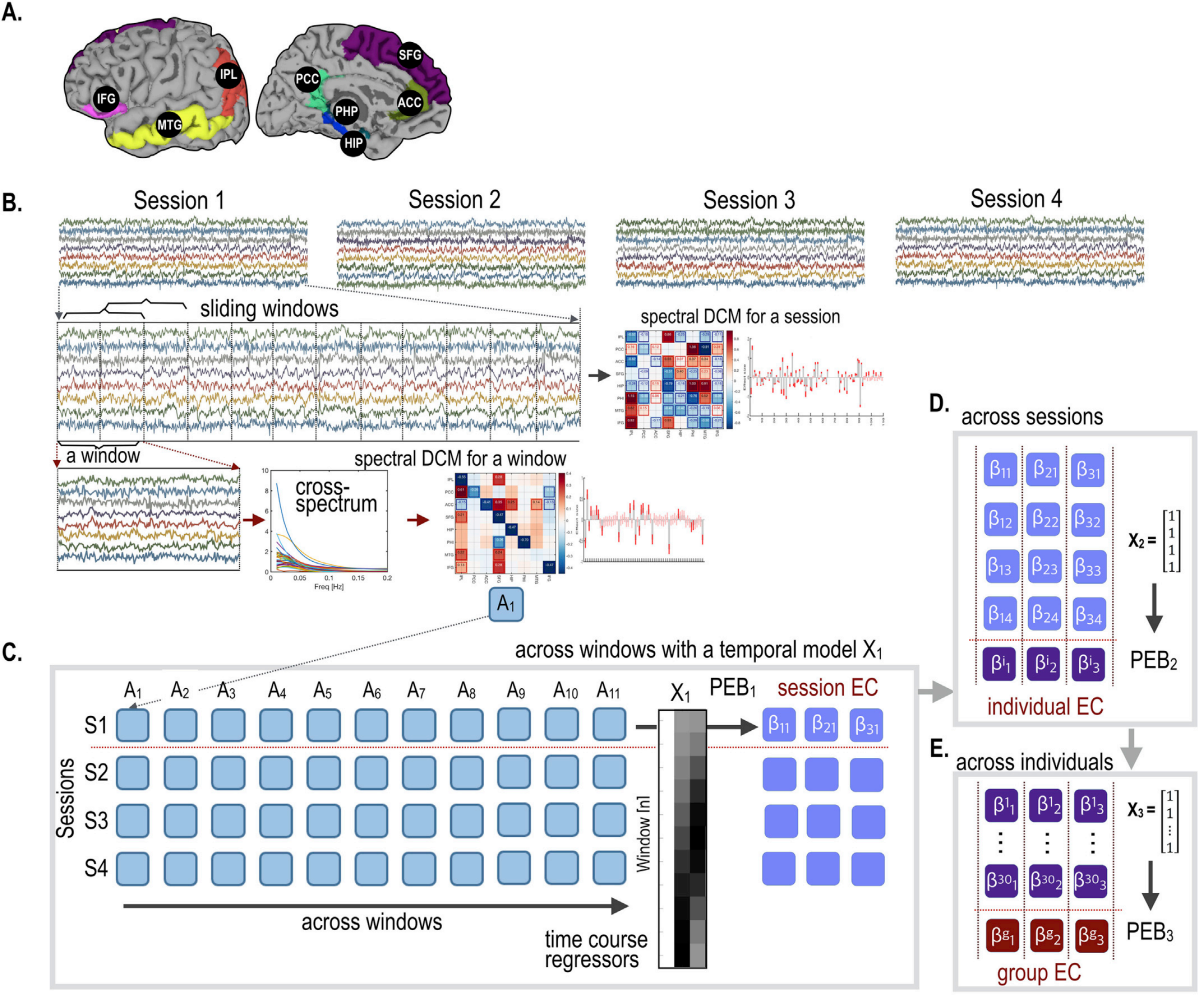
Procedures for dynamic effective connectivity (EC) analysis. The default mode network used in the current study is shown in (A). The default mode brain regions include the inferior parietal lobe (IPL), isthmus cingulate (PCC), rostral anterior cingulate gyrus (ACC), superior frontal lobe (SFG), hippocampus (HIP), parahippocampal gyrus (PHI), middle temporal gyrus (MTG) and pars orbitalis of the inferior frontal lobe (IFG) in the left hemisphere. Each of the four sessions of rsfMRI was subdivided into 11 overlapping windows (each with 200 time points) (B). A spectral DCM (spDCM) was estimated for each window separately for each session of every participant (C). Parametric empirical Bayesian analysis (PEB) with a temporal basis set was used to estimate baseline and dynamic components of effective connectivity in each session (PEB_1_). For the group level analysis, two additional steps of PEB analyses were applied; 1) PEB_2_ across four sessions with a column of constants ([1 1 1 1]^T^); 2) PEB_3_ across 30 participants with a column of [1 1 1 1 ⋅⋅⋅ 1 1]^T^ to model group effects.

The present study used the rsfMRI data of 30 participants from the HCP (15 males, ages: 29.3 ± 3.37 years). These participants were selected according to their ordering in the dataset. Non-selected participants had a history of neurological or psychiatric diagnosis, defined by DSM criteria and we excluded any twins. All data was sampled at TR = 0.72s, during four sessions, with 1200 time points per session. HCP rsfMRI data were preprocessed according to the HCP minimal preprocessing pipeline (Glasser et al., 2013). For the analysis of large-scale intrinsic brain networks, we extracted the rsfMRI time series from 8 regions of interest (ROIs), defined in a previously constructed automated labeling map (Desikan et al., 2006), corresponding to the default mode network (DMN) (Yeo et al., 2011). Those regions include the inferior parietal lobe (IPL), isthmus cingulate or posterior cingulate cortex (PCC), rostral anterior cingulate cortex (ACC), superior frontal gyrus (SFG), hippocampus (HIP), parahippocampal gyrus (PHP), middle temporal gyrus (MTG) and pars orbitalis of the inferior frontal lobe (IFG). To reduce computational cost, we restricted our analysis to brain regions in the left hemisphere (Fig. 1a). The first eigenvariate of each region was used as a regional BOLD signal summary.

### Spectral dynamic causal modelling (spDCM)

In the setting of rsfMRI, spDCM models endogenous fluctuations (in the absence of external input) using a state space model with two parts; a differential equation of neuronal dynamics and a haemodynamic response model *h*.

(1) $$\begin{array}{l} \dot{x}\left(t\right)=Ax\left(t\right)+v\left(t\right)\\ y\left(t\right)=h\left(x\left(t\right),\theta_{h}\right)+e\left(t\right),\;\;e\;∼\;N\left(0,\text{Σ}\right)\text{,} \end{array}$$

where *x*(*t*) represents a hidden neural state vector for brain regions at time *t* and the matrix *A* represents (intrinsic) effective connectivity among the regions. Endogenous or intrinsic (neural) fluctuations are denoted by *v*(t*)*. The measured BOLD signal *y* is modeled as a nonlinear haemodynamic response function *h* of neuronal states *x*(t) and parameters *θ*_*h*_ (based on the usual haemodynamic model (Stephan et al., 2007)) with an additive observation noise *e*(t).

To estimate the effective connectivity (i.e., the *A* matrix), Friston et al. (2014) proposed a Bayesian model inversion method in the spectral domain based on the observed cross-spectra – referred to as spectral DCM (spDCM). Here, we briefly summarized the procedure and the details can be found in Friston et al., 2014, Razi and Friston, 2016.

For summarizing observed cross spectra from the observed BOLD signal vector *y*(t), we used the Yule-Walker equation,

(2) $$y\left(t\right)=\sum_{i=1}^{p}a_{i}y\left(t-i\right)+z\left(t\right)$$

where $\left\{a_{1},\cdot \cdot \cdot ,\;a_{p}\right\}$ are MAR coefficients. The cross-spectra g(ω) of the BOLD signal can then be derived from the spectral density Y(ω),

(3) $$\begin{array}{l} \;Y\left(\omega \right)\;=A\left(\omega \right)\;\cdot \;Y\left(\omega \right)+Z\left(\omega \right),\;A\left(\omega \right)\\ \;\;\;\;=F\left(\left[a_{1},\cdot \cdot \cdot ,\;a_{p}\right]\right)\\ \;g\left(\omega \right)=\langle Y\left(\omega \right)\;\cdot \;Y{\left(\omega \right)}^{*}\rangle \end{array}$$

The generative model Eq. (1) can now be re-written as,

(4) $$\begin{array}{l} \;y\left(t\right)=k\left(\tau \right)⊗v\left(t\right)+e\left(t\right)\\ \;k\left(\tau \right)=\partial_{x}h\;\cdot \;\text{exp}\left(\tau \;\cdot \;\partial_{x}f\right)\text{,} \end{array}$$

where k(τ) is a Volterra kernel composed of neural state function *f* and haemodynamic response function *h*. $\partial_{x}f$ corresponds to the *A* matrix in the linear differential equation in Eq. (1). The autospectra of the endogenous fluctuations *v*(t) are usually modeled with a power law distribution, where the amplitude and exponents are parameterized by $\alpha_{v}$ and $\beta_{v}$ respectively. This choice is based on previous observations that the spectral content of the BOLD signal is “pink” or follows the scale free “1/f” distribution (Bullmore et al., 2001, He et al., 2010, Maxim et al., 2005):

(5) $$\begin{array}{l} g_{v}\left(\omega ,\theta \right)=\alpha_{v}\omega^{-\beta_{v}}+g_{u}\left(\omega ,\theta \right)\\ \;g_{u}\left(\omega ,\theta \right)=F(C\;\cdot \;u\left(t\right)) \end{array}$$

where F(.) denotes the Fourier transform and C denotes the weight matrix for the input *u*(*t*). If there are no external stimuli (u(t)=0), $g_{v}\left(\omega ,\theta \right)=\alpha_{v}\omega^{-\beta_{v}}$. The observation error can similarly be modeled as

(6) $$g_{e}\left(\omega ,\theta \right)=\alpha_{e}\omega^{-\beta_{e}}$$

In summary, the predicted cross-spectra of the BOLD signals, using Fourier transform of Eq. (3), can be written as

(7) $$\hat{g}\left(\omega ,\theta \right)=K\left(\omega \right)\;\cdot \;g_{v}\left(\omega ,\theta \right)\;\cdot \;K{\left(\omega \right)}^{*}+g_{e}\left(\omega ,\theta \right)$$

The observed cross spectra of bold signal g(ω) can then be considered as a noisy version of generative cross-spectrum $\hat{g}\left(\omega ,\theta \right)$

(8) $$g\left(\omega \right)=\hat{g}\left(\omega ,\theta \right)+N\left(\omega \right)$$

where N(ω) is sampling error.

The Bayesian model inversion of this generative model entails the estimation of posterior distribution of the model parameters, p(θ|g(ω),m), and the associated log-model evidence,  lnp(g(ω)|m). This inversion uses standard variational (Laplace) procedures that are described elsewhere (Friston et al., 2007, Razi and Friston, 2016).

### Dynamic spectral DCM

To characterize dynamic effective connectivity, we partition the time series into *W* windows, such that:

(9) $${\dot{x}}_{\left(i\right)}={\text{A}}_{i}x_{\left(i\right)}+v_{i}:i=1,\cdot \cdot \cdot ,W$$

(10) $${\text{ A}}_{i}={\text{A}}_{0}+{\text{A'}}_{i}={\text{A}}_{0}+\sum_{k=1}^{K}{\text{A}}_{\left(k\right)}X_{k}\left(i\right)\;$$

A_*i*_ is the effective connectivity for the *i*-th window, which can be decomposed into (i) a baseline component ${\text{A}}_{0}$ that is conserved over windows and (ii) a dynamic component ${\text{A'}}_{i}$ that varies with each window. ${\text{A'}}_{i}$ can be modeled with a combination of *K* temporal basis functions *X*_(*k*)_(*i*) and their corresponding effective connectivity matrices A_(*k*)_. Under this model, it is now necessary to estimate A_0_ and A_(k)_, for which we use PEB, where the second (between-window) model is specified in terms of the temporal basis functions.

### PEB estimation of dynamic effective connectivity

To estimate baseline and dynamic intrinsic effective connectivity components, we modeled between-window fluctuations in spDCM estimates of within window effects, using a hierarchical Bayesian approach with SPM12 (Friston et al., 2015, Friston et al., 2016). We inverted spDCMs separately for each time-window of rsfMRI data for each session at the first (within window) level. The first level spDCMs for all windows in a session were then modeled at the second (within-session, between-window) level using a PEB scheme (Friston et al., 2016). More specifically, at the first level, the intrinsic connectivity matrix *A* was specified as a fully connected graph. The second level comprised a linear model with session effects $\beta_{j}^{\left(2\right)}\;$ encoded by a between-window design matrix, X_1_. The implicit hierarchical generative model can be summarized as follows:

(11) $$g{\left(\omega \right)}_{ij}=\text{Γ}\left(\theta_{ij}^{\left(1\right)}\right)+\varepsilon_{ij}^{\left(1\right)}:\;\;\varepsilon_{ij}^{\left(1\right)}\;∼\;N\left(0,{\text{Σ}}^{\left(1\right)}\right)$$

(12) $$\;\theta_{ij}^{\left(1\right)}=X_{1}\beta_{j}^{\left(2\right)}+\varepsilon_{j}^{\left(2\right)}:\;\;\;\;\;\varepsilon_{j}^{\left(2\right)}\;∼\;N\left(0,{\text{Σ}}^{\left(2\right)}\right)\text{,}$$

where Γ stands for the prediction of observed cross spectra for the *i*-th window based on parameters $\theta_{ij}^{\left(1\right)}$ sampled from the *j-*th session-level average (across-windows) with a random effect $\varepsilon_{j}^{\left(2\right)}$. The between-window design matrix, $X_{1}$, comprised the temporal regressors $\left[{\text{X}}_{\left(0\right)}\;{\text{X}}_{\left(1\right)}\;{\text{X}}_{\left(2\right)}\cdot \cdot \cdot \;{\text{X}}_{\left(K\right)}\right]$ in Eq. (7) where $X_{\left(0\right)\;}$ is a column vector of ones that models baseline effective connectivity over windows. In this form, the second level parameters $\beta_{j}^{\left(2\right)}\;$ contain the elements of the dynamic effective connectivity ${\text{A}}_{\left(k\right)}$ in Eq. (5).To ensure robust estimation of first level effects $\theta_{ij}^{\left(1\right)}$, we iteratively estimated the posterior parameter distribution (using **spm_dcm_peb_fit.m** in SPM12), using the session means as empirical prior (Friston et al., 2015, Friston et al., 2016, Litvak et al., 2015).

Each effect of interest (i.e., $\beta_{j}^{\left(2\right)}\;$ corresponding to the regressors in the second level design matrix) for each session was evaluated with respect to inter-session variation at the third level using a between-session design matrix, $X_{2}$ (please see Fig. 1D).

(13) $$\beta_{j}^{\left(2\right)}=X_{2}\beta^{\left(3\right)}+\varepsilon^{\left(3\right)},\;\;\varepsilon^{\left(3\right)}\;∼\;N\left(0,{\text{Σ}}^{\left(3\right)}\right)$$

To estimate average connectivity (across sessions) in each individual, we used $X_{2}$ = [1 1 1 1]^*T*^.

Finally, for group level analysis across subjects, we conducted an additional level of PEB (Fig. 1E): PEB of individual effect sizes (i.e., average effective connectivity across sessions for each individual) across individuals, with a design matrix X_3_ for group inference (i.e., PEB of PEB). The group averages across individuals were estimated by using a design matrix X_3_ = [1 1 1 1 … 1]^*T*^ for N subjects. Note that this recursive application of PEB is very similar to the standard summary statistic approach for mixed effects models in classical inference. The key difference here is that we take both the posterior expectations and covariances from one level to the next to assimilate data in a Bayesian fashion.

### Estimating dynamic effective connectivity using temporal basis functions

We modeled baseline and dynamic effective connectivity using PEB with four different types of basis sets in the second level design matrix ($X_{1}$): (i) a vector of ones for a simple average across windows, (ii) a discrete cosine transformation (DCT) basis set, (iii) a basis set based on principal component analysis (PCA) of first level *A* matrices and (iii) a basis set based on functional principal component analysis (fPCA) of first level *A* matrices: we included fPCA as a dynamic model to incorporate temporal smoothness. In more detail:

Model 1 Simple average model:

In this model, we assumed effective connectivity can be modeled as a random variation around the average effective connectivity, with a Gaussian distribution. This average model can be specified with the design matrix:

$$X_{1}={\left[1\;1\;1\;1\;\cdots \;1\right]}^{T}$$

Model 2 DCT based model:

DCT functions (CS) at the second level, model systematic fluctuations in effective connectivity and can be constructed as:

(14) $$X_{1}=\left[CS_{0}\;CS_{1}\;CS_{2}\cdots CS_{W-1}\right]$$

where $CS_{k}$ is the column vector composed of $\sqrt{\frac{2}{N}}\;\cos\left(\frac{\pi }{N}\left(n+\frac{1}{2}\right)k\right),\;n=0,\cdots ,W-1.$

Model 3 PCA based model:

In this model, we used PCA of the effective connectivity matrices A from the first level to extract eigenvariates (or modes) of first level effective connectivity. The principal component transformation was based upon the singular value decomposition (SVD) of successive (vectorised) connectivity estimates and the principal singular variates were used as second level regressors.

Model 4 fPCA based model

In order to ensure temporally smooth eigenvariates, we also used functional principal component analysis (fPCA) (Li et al., 2016). This supervised fPCA is derived by fitting a model of the form:

(15) $$X=UV^{T}+E,\;U=YB+F$$

where *X* is a data matrix (successive estimates of vectorised *A* matrix), *U* is a latent score matrix, *V* is a loading matrix, *E* is observed noise, *Y* is an observed auxiliary supervision matrix, *B* is a coefficient matrix, and *F* is a random effect matrix. The data matrix *X* can be decomposed into low-rank components, while accounting for supervision by any auxiliary data *Y* measured on the same samples. This model also counts for smoothness and sparsity in supervision coefficients *B*. For details, see (Li et al., 2016). Here in this study, we only used the smoothness constraints without enforcing a sparsity structure.

All of these models comprised 6 (or less) temporal basis functions, where the first column of the design matrix is a vector of ones, modelling a session average or baseline effective connectivity and subsequent columns model dynamic components. Finally, we compared these models of dynamic effective connectivity with a conventional method that estimates the connectivity using the entire time-series without any windowing – referred to as the ‘stationary model’.

We used the above approach to characterize the functional integration of DMN. The DMN contained 8 nodes that included the inferior parietal lobe, isthmus cingulate, rostral anterior cingulate gyrus, superior frontal lobe, hippocampus, parahippocampal gyrus, middle temporal gyrus and pars orbitalis of the inferior frontal lobe in the left hemisphere. Each session of 1200 time points sampled in those regions was segmented into 11 overlapping windows (with a window size of 200 time points and an overlap of 100 time points).

Fig. 1 illustrates procedures for estimating dynamic effective connectivity in each session – and the procedure to assimilate effective connectivity estimates at the group level using multi-level or recursive PEB.

**Fig. 2:**
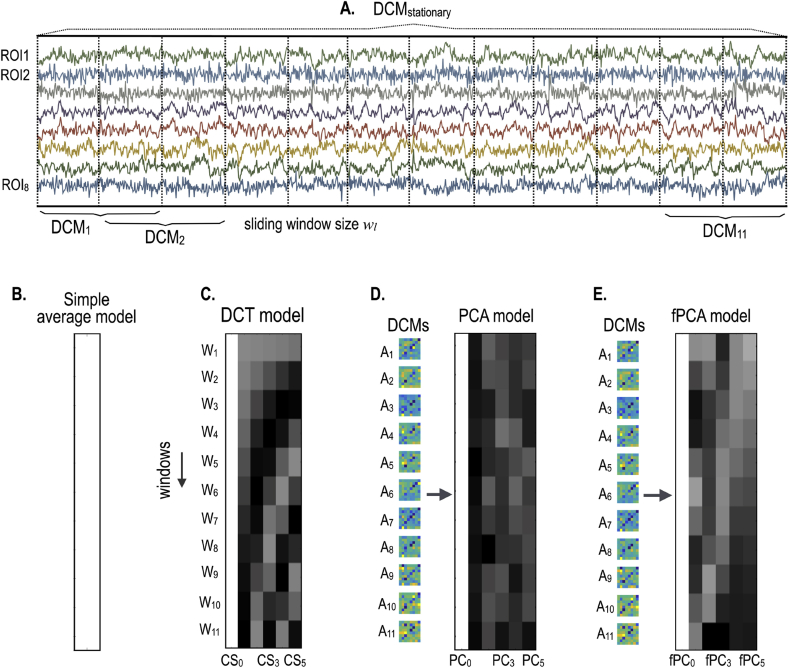
Second level models for dynamic effective connectivity analysis. For the whole time series of the default mode brain regions (ROI_1_,⋅⋅⋅, ROI_8_), a spDCM was estimated under the assumption of the stationarity during the session (A). To characterize the dynamics of the intrinsic effective connectivity, we constructed four types of second level models; a simple average model, i.e., Bayesian average of DCMs for all windows (B), a DCT model with DCT basis function as regressors (C), PCA (D) and fPCA models (E) that were based on the initial estimates of intrinsic effective connectivity.

Fig. 2 illustrates the models used in the current study; i) a stationary model, ii) a simple average model, iii) a DCT-based model, iv) a PCA model and v) a fPCA model of second level effects.

### Face validation of dynamic effective connectivity using simulations

To assess the face validity of the proposed scheme, we conducted a simulation study with known fluctuations in effective connectivity among four nodes. The baseline and dynamic effective connectivity matrix – used for generating BOLD signals – were based on Eq. (9) and Eq. (10); using biologically plausible parameters from spDCM estimates of real rsfMRI data. The BOLD time series was simulated using a standard (nonlinear) hemodynamic response function (Stephan et al., 2007). Specifically, the matrices in Eq. (10) used to generate synthetic BOLD data were:

$${\text{A}}_{0}=\left[\begin{array}{cccc} -0.5289 & -0.1243 & 0.2943 & 0.0534\\ -0.1795 & -0.7139 & -0.0158 & 0.0012\\ 0.1032 & 0.1848 & -0.6805 & -0.1134\\ 0.1824 & 0.0514 & -0.0577 & -0.8405 \end{array}\right]$$

$${\text{A}}_{\left(1\right)}=\left[\begin{array}{cccc} 0 & 0.2884 & -0.2666 & -0.0841\\ 0.1784 & 0 & 0.0432 & -0.1235\\ -0.1815 & -0.2779 & 0 & 0.0058\\ 0.0967 & 0.2091 & -0.0180 & 0 \end{array}\right]$$

(16) $${\text{A}}_{i}={\text{A}}_{0}+{\text{A}}_{\left(1\right)}X_{1}\left(i\right)\;:\;i=1,\cdot \cdot \cdot ,W$$

$$X_{1}\left(i\right)=[\begin{array}{c} \;\;\;\;\;\;\;0\;\;\;\;\;\;\;\;\;\;\;\;\;\;\;\;\;\;\;\;\;\;\;\;\;\;\;\;\;\;\;\;\;\;\;\;\;\;\;\;\;\;\;\;\text{for a stationary simulation}\\ \sqrt{\frac{2}{N}}\;\cos\left(\frac{\pi }{W}\left(i+\frac{1}{2}\right)\right)\;\;\;\;\;\;\;\;\;\;\;\;\text{for a dynamic simulation} \end{array}$$

First, we generated a stationary time series with 3000 time points (scans), with a TR = 0.72sec using A_0_ only. To generate dynamic (nonstationary) time series (3000 scans with a TR = 0.72sec) we concatenated fifteen sliding windows (each of 200 scans). The time series for each window was generated using dynamic effective connectivity ${\text{A}}_{i}\;$ modeled according to Eq. (16). For 15 non-overlapping windows of size 200, we conducted a dynamic effective connectivity analysis with two DCT basis functions in the second level design matrix X_1_: see Eq. (14). These between window regressors comprised a vector of ones (modelling stationary effects) and the first cosine function (modelling dynamic effects). We then compared 2-s level models (stationary and dynamic) for the two types of synthetic data (stationary and dynamic), using Bayesian model reduction and comparison (**spm_dcm_bmc_peb)** (Friston et al., 2016). For the inversion of DCM for each window, we used the estimated DCM parameters of the first window as a prior for the second level model across windows using **spm_dcm_peb_fit**. In summary, we generated data with and without fluctuations in effective connectivity and then inverted models, with and without dynamic effective connectivity, to ensure we could infer the presence of dynamic fluctuations when they were present – and their absence when they were not.

**Fig. 3:**
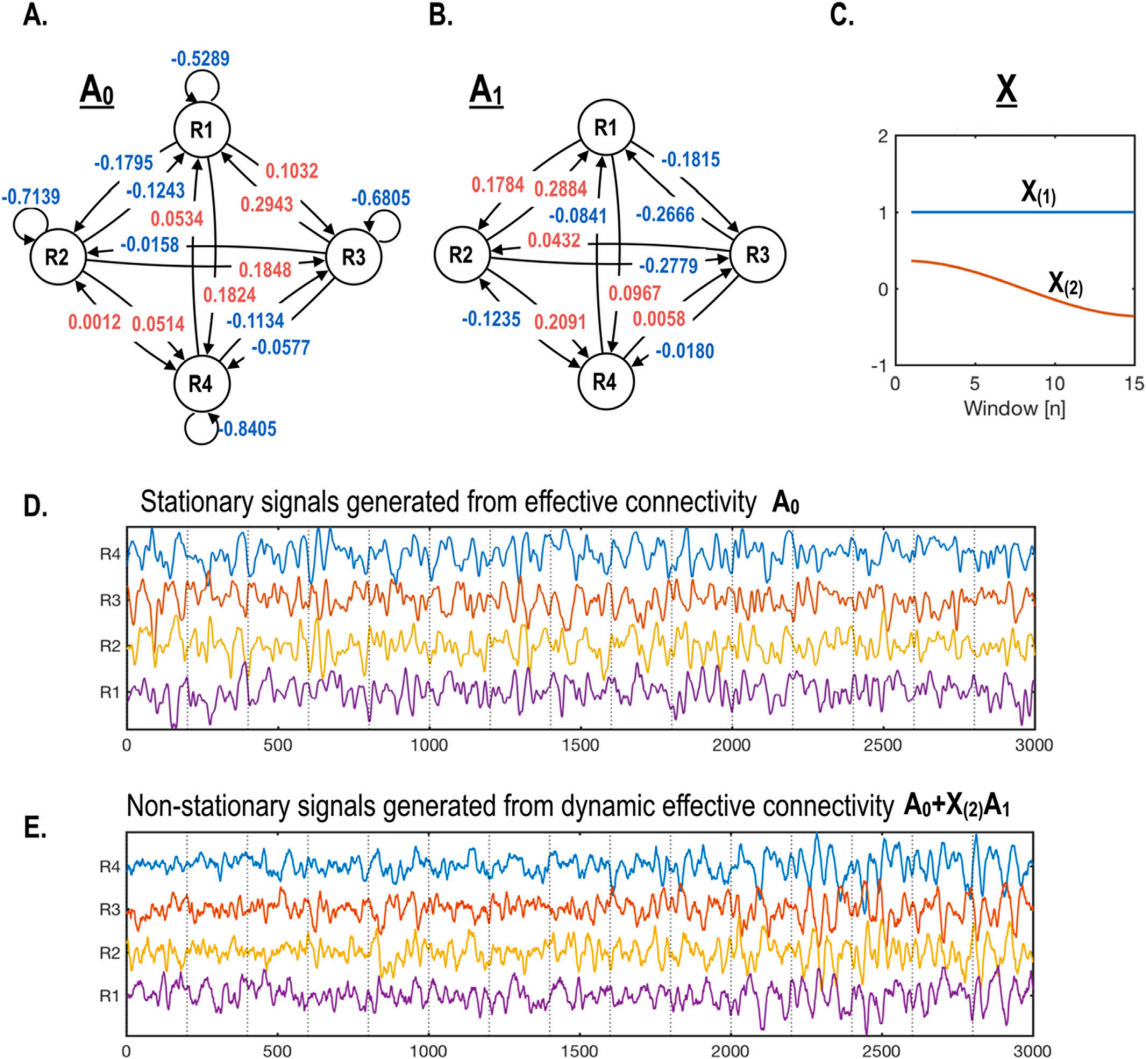
Simulating dynamic effective connectivity. (A) and (B) described the baseline and dynamic components of intrinsic effective connectivity (A_0_ and A_1_) used for generating synthetic data. Stationary time series (D) were generated using the baseline effective connectivity A_0_ only. To simulate dynamic nonstationary time series, we used a second level design matrix X with a column of ones and a cosine function X_(2)_. For each window, a baseline effective connectivity matrix (A_0_) was combined with a dynamic component (A_1_) – weighted by the cosine function X_(2)_ – to generate a dynamic time series (E).

Fig. 3 shows the four-node simulation setup. We used baseline connectivity A_0_ (as shown in Fig. 3A) to generate stationary time series (Fig. 3D), while the nonstationary time series in Fig. 3E was generated using both baseline (A_0_) and dynamic (A_1_) components of the effective connectivity (as shown in Fig. 3A and B). Fig. 3C shows the design matrix used to generate dynamic effective connectivity for each window according to Eq. (16).

**Fig. 4:**
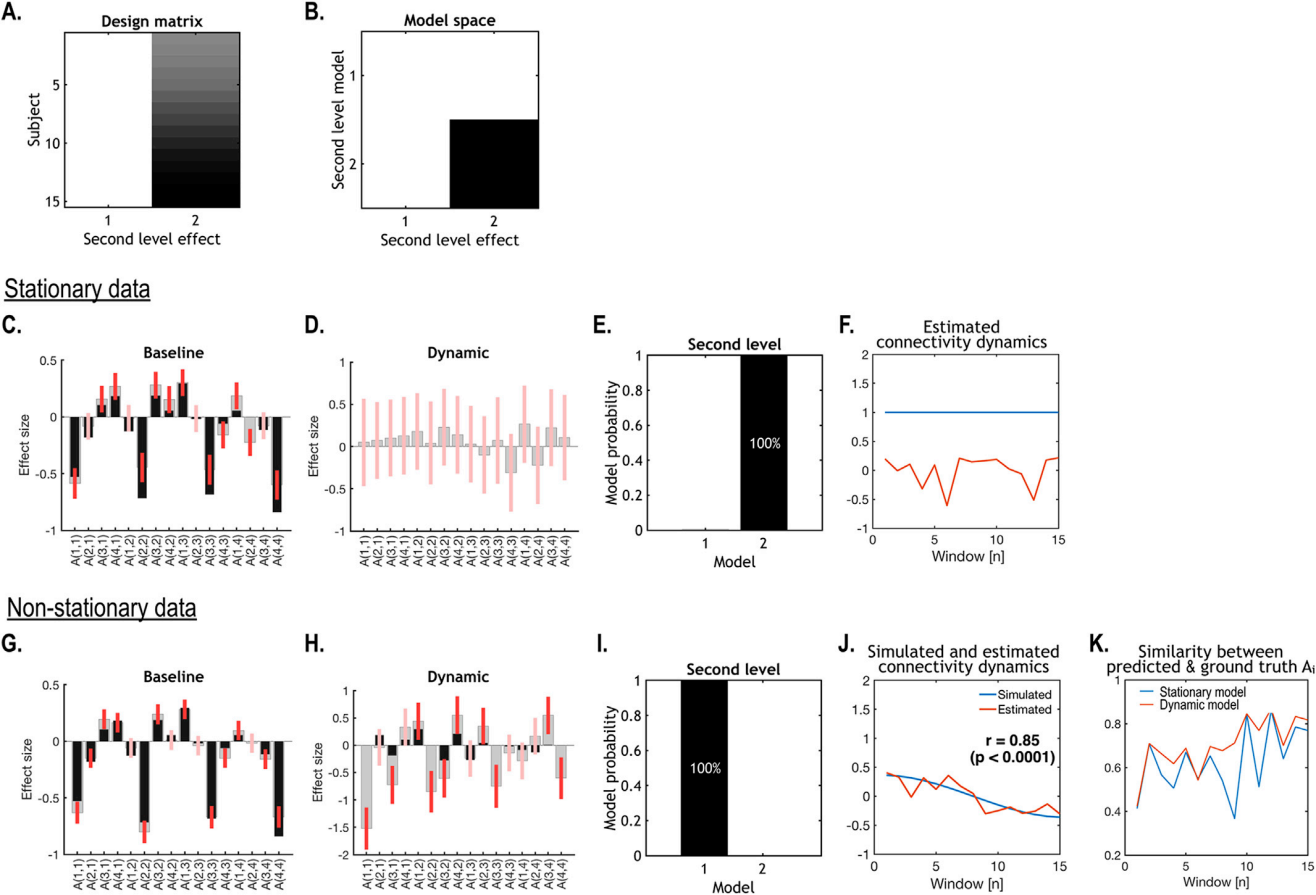
Simulation results of dynamic effective connectivity analysis of the BOLD regional time series presented in previous figure. We applied Bayesian model inversion to the stationary and nonstationary time series in Fig. 3, using the same second level design matrix (A), followed by a Bayesian model comparison of two models (B). For stationary and nonstationary time series, we estimated a baseline component of the effective connectivity (C and G) corresponding to the first column of the design matrix (A) and a dynamic component (D and H) corresponding to the second column of the design matrix. In C, D, G and H, the grey and black bars indicate estimated and ‘true’ (i.e. values used to generate data) effective connectivity respectively for each connection. The red bars indicate 95% confidence intervals around the posterior estimates (grey bars). The leading diagonal of the A matrix parameterises the log-scaling a negative (−0.5 Hz within-region) self-connection in DCM for fMRI. Thus, negative diagonal elements of A indicate disinhibition; i.e., a self-inhibition closer to zero. Similarly, fluctuations in self-connections reflect fluctuations in log-scaling. For stationary time series, Bayesian model comparison returned a higher posterior probability for the model with only a baseline component (the first column in B) compared to the model with both baseline and dynamic (the second column in B) components (E). For nonstationary time series, Bayesian model comparison showed that the model with both baseline and dynamic components wins over the model with only a baseline component (I). We applied PCA to the dynamic effective connectivity to retrieve the estimated dynamics of the first principal component (after removing the average) (J). The ensuing principle eigenvariate was very similar to connectivity used to generate data (C in Fig. 3) (r = 0.8536, p = 0.000). The similarity between the ‘ground-truth’ effective connectivity A_i_ and the effective connectivity predicted by the dynamic second-level model (Model 1) at each window was generally higher than that for the stationary model (Model 2) (K).

Fig. 4 shows the simulation results after estimating dynamic effective connectivity and Bayesian model comparison of the two models (i.e., stationary versus dynamic) to establish the face validity of the procedure. This example shows that the evidence for the stationary model (in terms of its posterior probability) was higher than that of the dynamic model for a stationary time series (Fig. 4C–F). Conversely, the evidence for the dynamic model was higher than that of the stationary model for the nonstationary time series (Fig. 4G–J). In summary, given typical data generated under ideal stationary and nonstationary conditions, Bayesian model comparison was able to recover the correct model. Interestingly, the dynamic model provided slightly more accurate estimates – in relation to ground truth effective connectivity – than the stationary model, at each window (Fig. 4K).

### Evaluation of dynamic effective connectivity of DMN

In order to evaluate the estimates of dynamic effective connectivity, we used the criteria of stability of baseline effective connectivity across sessions. We hypothesized that effective connectivity is dynamic within and between sessions but baseline effective connectivity *A*_0_ estimated from multiple windows at each session would be conserved across sessions. Accordingly, we expected that the baseline *A*_0_ would be less variable across the four sessions than stationary connectivity *A* (using the entire time series in a session). In order to test this hypothesis, stability was defined as the average cross-correlation between *A*_0_ matrices across the four sessions. For the five models (one stationary and the four models that characterize dynamics using temporal basis sets) in 30 individuals, we conducted one way analysis of variance (ANOVA) with repeated measures of group-level similarities of *A*_0_ across sessions. For the stationary model, *A* was used in place of the baseline *A*_0_, of dynamic connectivity models.

We performed Bayesian model inversion of the full model at the second level followed by Bayesian model reduction to find the best reduced (nested) model (using **spm_dcm_bmc_peb.m)**. In principle, one can perform Bayesian model comparison at two levels; at the first that distinguishes between different architectures at the level data are generated and at the second, where we specified explanatory variables at the between-window level (i.e., systematic fluctuations in dynamic effective connectivity). However, in order to test for the optimal combinations of temporal basis functions (i.e. DCT, PCA and fPCA), we limited model comparison to the second level by scoring the reduced models (using log-model evidence or its proxy free energy). Our focus here was on identifying the optimal number of temporal basis functions within each model. In other words, to estimate the number of dynamic effective connectivity modes that could account for the implicit dynamic functional connectivity.

### Multilevel PEBs for group level DMN connectivity estimation

To construct a group-level model of DMN intrinsic connectivity, we applied PEB recursively (see Eq. (7) and explanation above); i) PEB across windows, ii) PEB across sessions and iii) PEB across subjects. PEB across windows was used to derive dynamic effective connectivity as explained above. After estimating dynamic effective connectivity for each session of each individual, we averaged four sessions in each individual by using across session PEB, followed by across subject PEB of across session PEB results over 30 individuals.

## Results

**Fig. 5:**
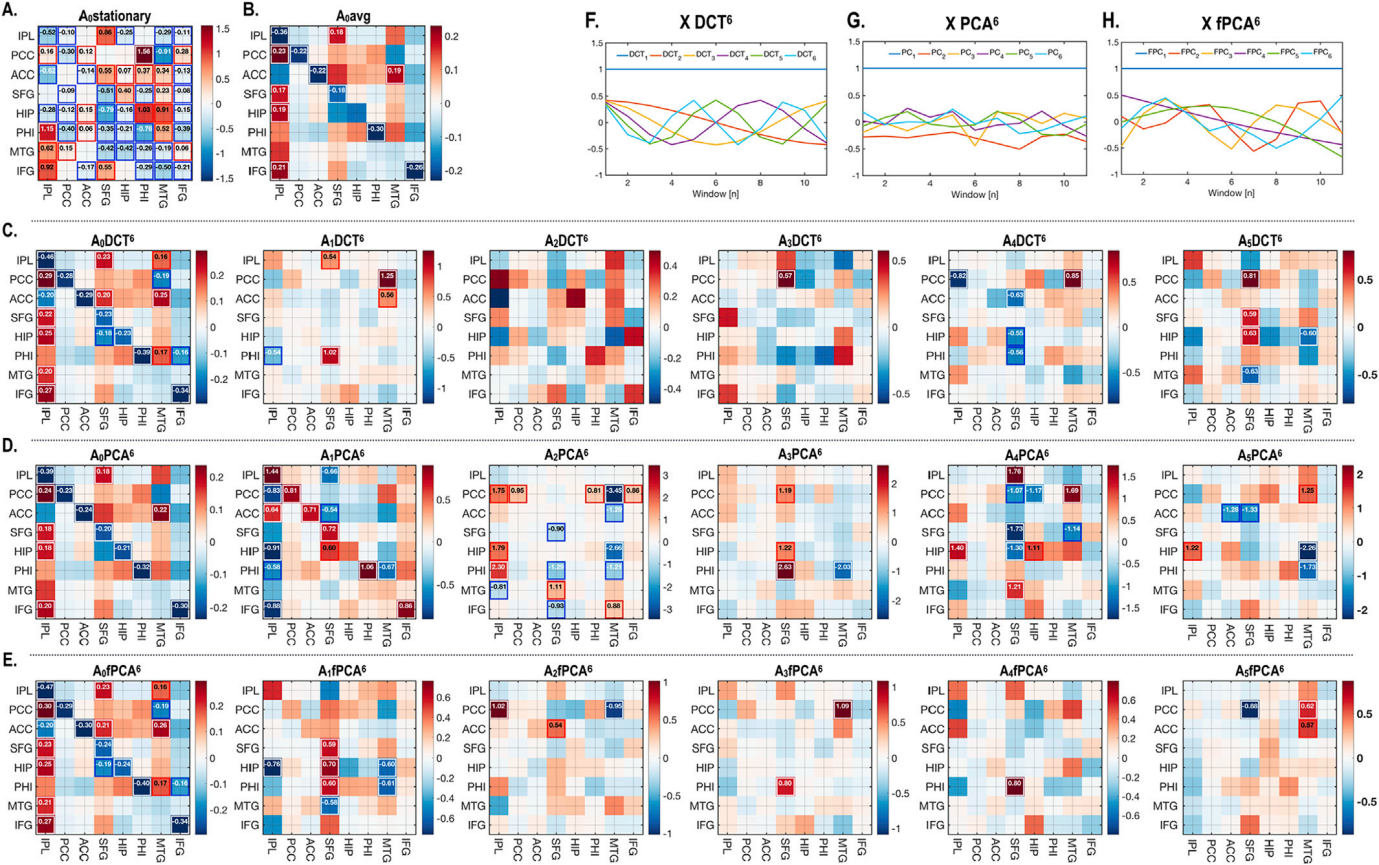
Baseline and dynamic effective connectivity components of the default mode network during a session of an exemplar participant. The intrinsic effective connectivity for whole time series under stationarity assumption (*A*_0_stationary) is displayed in (A). (B) reports the average model (*A*_0_avg) of all windows in the session. The dynamic components of effective connectivity are displayed according to the temporal basis function model; DCT (C), PCA(D) and fPCA (E). All models have a design matrix with up to the 6th temporal basis (among total 11 bases), denoted with a superscript for each model. The first regressor is a vector of ones. Effect sizes that survived a criterion of 95% posterior confidence, or more, are shown in rectangles. The basis sets or design matrices used to model the dynamics across windows are displayed in (F) for DCT, (G) for PCA and (H) for fPCA. The adjacency matrix element *A*(i,j) can be interpreted as effective connectivity from the column to row. For better illustration purpose, the colors in the adjacency matrix was trimmed at two standard deviations deviated from the average of all elements in the adjacency matrix.

**Fig. 6:**
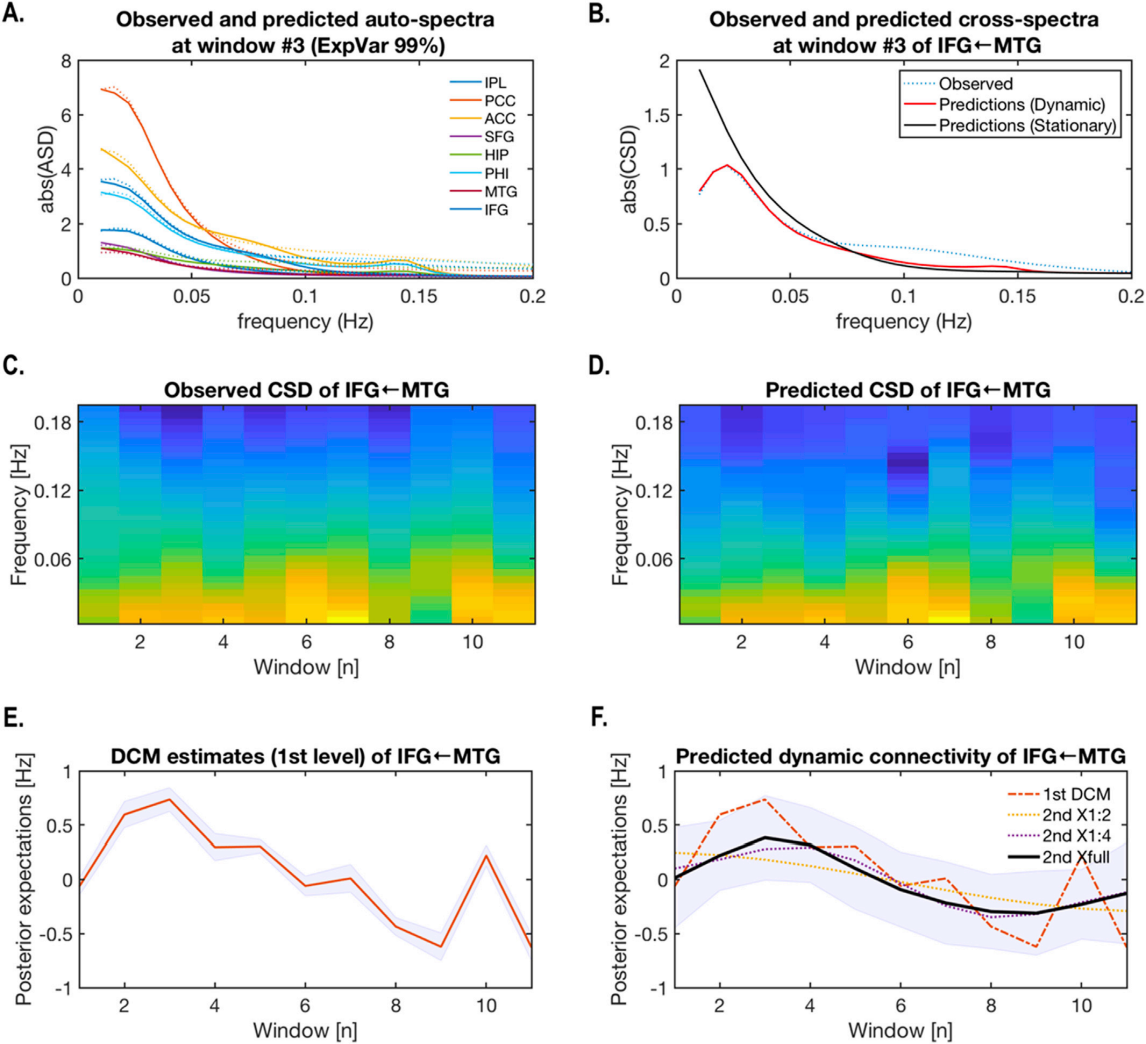
An example of dynamic effective connectivity estimation. (A) Predicted (solid lines) and observed (dotted lines) auto-spectra (ASD) of eight nodes of the default mode network are shown. (B) For the connection from MTG to IFG, predicted CSD with a dynamic model (red line) was very similar to the observed CSD (dotted line) for temporal window (#3) but differed significantly from the averaged CSD, across windows, in a stationary model (black line). (C) and (D) present the dynamics of both observed and predicted CSDs of the connection from MTG to IFG, which was estimated at the first level. Color levels indicate log-transformed CSD powers. (E) presents a time course of effective connectivity estimates (posterior expectations as the red line and confidence intervals in grey) of the same connection from MTG to IFG, with the window index on the x-axis. (F) shows the time course (black line) of effective connectivity of the same connection (IFG ← MTG) but estimated at the second level with a full (six basis functions) design matrix of DCT^6^ (2nd Xfull). (F) reproduces the slow fluctuations (dotted lines) in the first level DCM estimators (in E), time courses of predicted connectivity with the first and second DCT basis function (2nd X1:2), and time courses of predicted connectivity with (up to) four DCT basis functions (2nd X1:4).

**Fig. 7:**
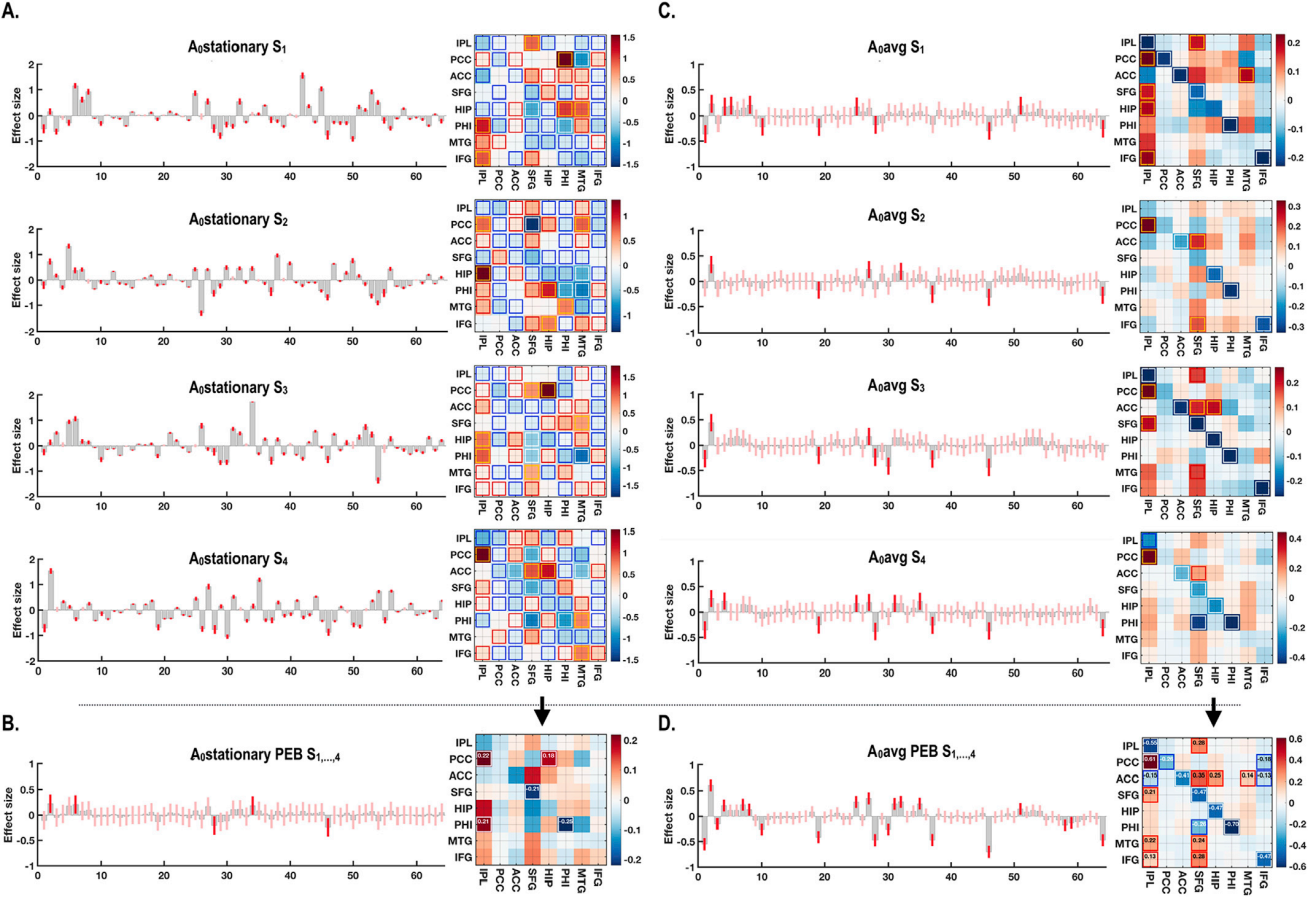
An exemplary dynamic effective connectivity of four sessions (S_1_, S_2_, S_3_ and S_4_), from the same participant in Fig. 5 (A) Stationary effective connectivity (for the complete time series in a session, i.e., A_0_stationary) and (B) PEB average of stationary effective connectivity across sessions. (C) Average effective connectivity under a simple average model (A_0_avg) for each session and (D) its PEB average across sessions are shown. PEB average of A_0_stationary reduced statistical significance (indicating variations across sessions) while PEB average of A_0_avg increased the statistical significance, showing consistency across sessions). Effect sizes that survived a criterion of 95% posterior confidence, or more, are shown in rectangles.

Fig. 5, Fig. 6, Fig. 7 illustrate an exemplary individual analysis. According to the second level model, dynamic effective connectivity evolves according to the temporal basis functions of each model (Fig. 3). Fig. 5 shows the baseline and dynamic components corresponding to each model. We have indicated effective connectivity that survived a non-zero criterion with a posterior confidence of 95% (using rectangles); i.e., when the confidence interval does not contain zero. Note that fPCA models slower fluctuations in connectivity compared to PCA (Fig. 5G and H).

Fig. 6 presents an exemplary case of dynamic cross-spectral densities (observed and predicted) and parameters estimated by the dynamic model using the data from the same participant presented in Fig. 5. The cross-spectral density (CSD) was predicted with high accuracy when spectral DCM was applied to each window (Fig. 6A) and was better predicted under a dynamic model, in relation to a stationary model (Fig. 6B). Note that the CSD fluctuates across windows (Fig. 6C) as a result of changes in effective connectivity (Fig. 6E). Fig. 6F shows that using all the basis functions approximates the effective connectivity estimated at each window, compared to any other combination of basis functions. The predicted time course of effective connectivity (Fig. 6F) suggests that the hierarchical model can exploit slow fluctuations in effective connectivity over time windows to provide empirical priors on first level (within window) estimates. These (empirical) prior constraints underwrite the rationale for the proposed approach to modelling dynamic changes in effective connectivity.

Fig. 7 illustrates cross-session consistency of dynamic effective connectivity within a participant. As shown in Fig. 7A, the effective connectivity based on the complete time series (without windowing) within a session under stationary model (A_0_stationary) showed many significant effective connections for each session. However, the intrinsic connectivity varied across sessions and most of the significant connections disappeared when averaged (using PEB) over the four sessions (Fig. 7B). In contrast, baseline effective connectivity (A_0_avg) under models of dynamic effective connectivity were relatively consistent across sessions and showed a greater number of significant connections when averaged, using PEB, across (four) sessions (Fig. 7C and D).

**Fig. 8:**
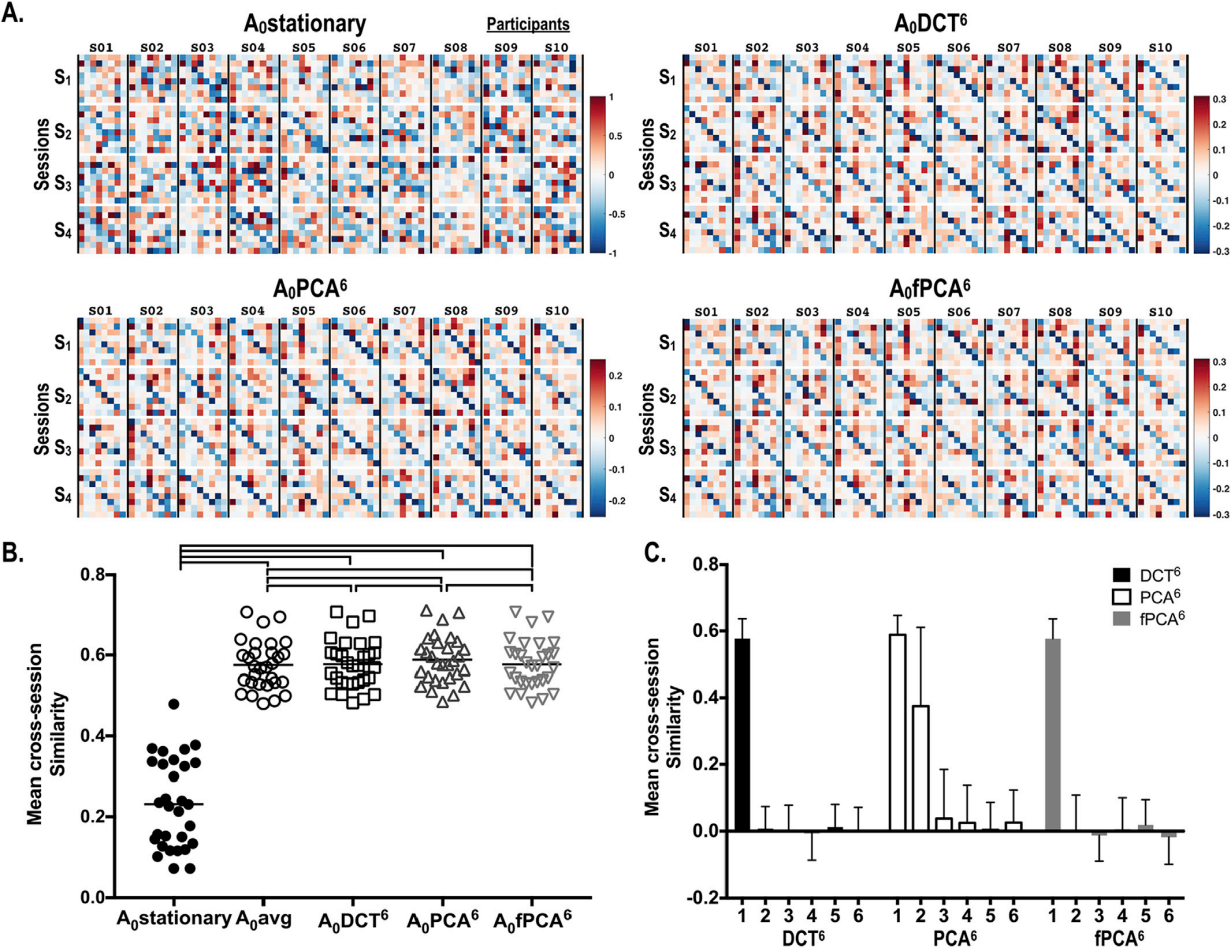
Intrinsic effective connectivity of the default mode network shown for each of the four sessions for 10 participants (A) and cross-session similarities of the baseline intrinsic effective connectivity *A*_0_ among 30 participants for several temporal basis sets (B); 1) a stationary model (A_0_stationary, mean ± standard deviation 0.234 ± 0.110), 2) a simple average model (*A*_0_avg, 0.575 ± 0.060), 3) a DCT (up to 6th component) model (*A*_*0*_DCT^6^, 0.577 ± 0.060), 4) a PCA (up to 6th principal components) model (*A*_*0*_PCA^6^, 0.588 ± 0.059) and 5) a fPCA (up to 6th functional components) model (*A*_*0*_fPCA^6^, 0.577 ± 0.060). The upper lines between models in (B) indicate significant between-group difference (p < 0.001, Bonferroni corrected). The mean cross-session similarities (among 30 participants) are displayed for the three temporal bases sets we used (C). Here the first component corresponds to the baseline effective connectivity *A*_0_ at the second-level.

Fig. 8A reports examples of baseline effective connectivity *A*_0_ across sessions in 10 participants. We found higher variation across sessions in the effective connectivity of the stationary model (A_0_stationary). In contrast, the baseline effective connectivity (*A*_0_) under all dynamic models showed greater consistency across sessions. This is clearly seen using the mean cross-session similarity of the baseline effective connectivity *A*_0_ in Fig. 8B. Compared to the connectivity estimate under stationarity assumptions (A_0_stationary), the baseline connectivity under non-stationary (dynamic) models was highly consistent across sessions in every participant (Fig. 8B).

A one-way repeated measures ANOVA shows a significant main effect of models on the cross-session similarity (F(1.011, 29.32) = 362.9, p < 0.0001). Post hoc comparisons using Bonferroni correction indicated that cross-session similarity at A_0_stationary condition (mean ± standard deviation, 0.234 ± 0.110) was significantly lower than all of non-stationary conditions; *A*_0_avg (0.575 ± 0.060), *A*_0_DCT (0.577 ± 0.060), *A*_0_PCA (0.588 ± 0.059) and *A*_0_fPCA (0.577 ± 0.060). The cross-session similarity of *A*_0_avg was significantly lower than that of *A*_0_ with temporal regressors (*A*_0_DCT, *A*_0_PCA, and *A*_0_fPCA) (p < 0.001). *A*_0_PCA had highest cross-session similarity among the models evaluated in the current study (Fig. 8B).

As shown in Fig. 8C, the cross-session consistency of the dynamic components is very low, except for the second PC component. In the PCA model, the first baseline component and the second (dynamic) component were consistent over sessions; although the across-subject variation of this similarity was high for the second component.

**Fig. 9:**
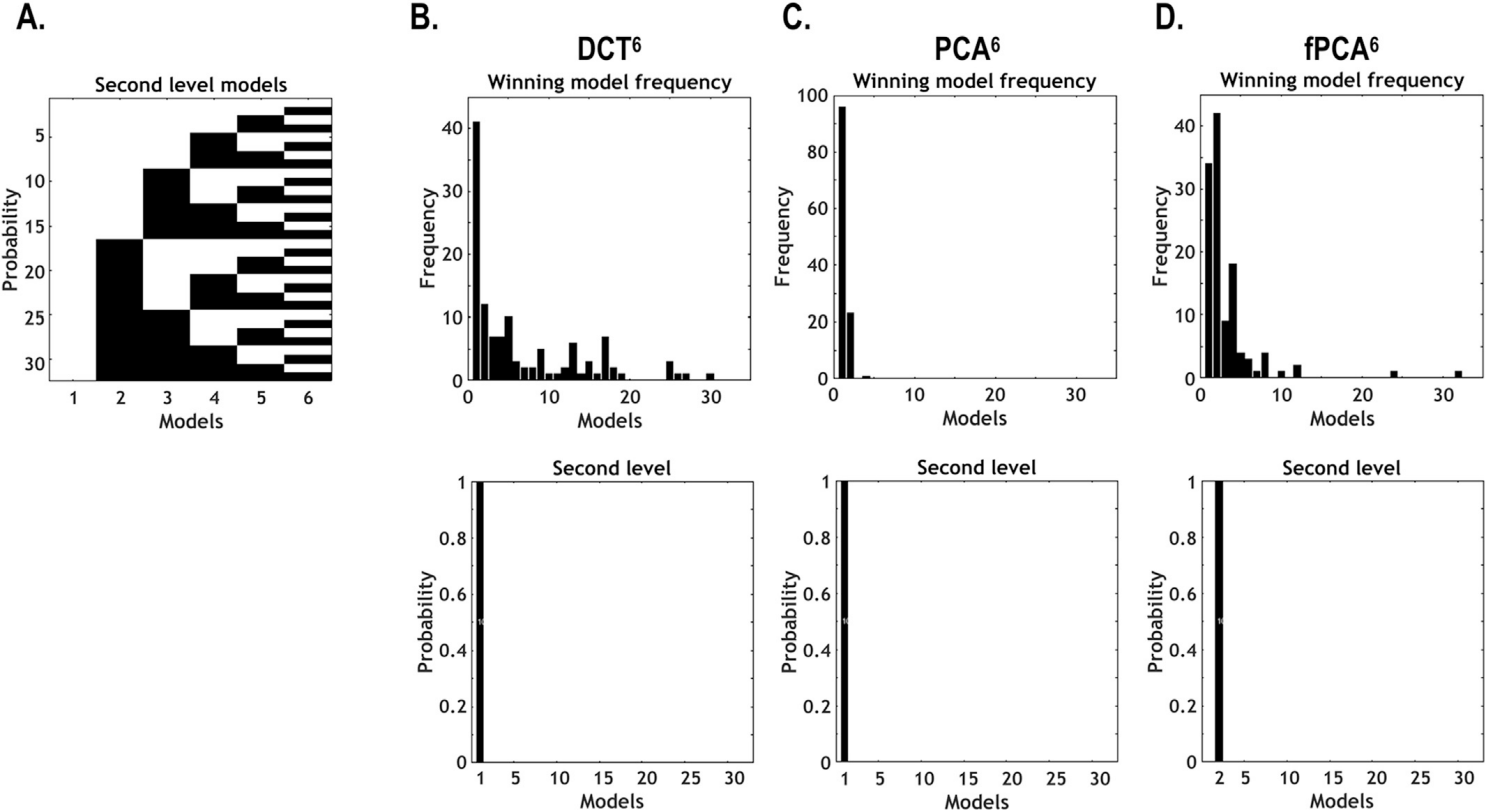
Bayesian model comparison for combinations of regressors at the second level (i.e., second-level models) with different temporal basis sets. We constructed 32 models with different combinations of six regressors (in the second-level, between-window design matrix X^1^, corresponding to six temporal basis functions) for three types of temporal basis sets, e.g., DCT^6^, PCA^6^ and fPCA^6^ (A). The upper panels of (B), (C) and (D) indicate the frequency of winning models for DCT, PCA and fPCA and the lower panels indicate the posterior probability for each model. Model comparison (among 32 combinations of six temporal basis functions for each type of temporal basis set) was performed for 120 sessions (four sessions each for 30 participants). In the current example, models with all six basis functions (for every type of basis sets) supervene over the other models with any alternative combination of temporal basis functions (except for fPCA6).

Fig. 9 reports model comparison for the second level temporal basis functions. For each of the six regressors (one constant and five dynamic bases), 32 combinations of regressors (including the first regressor) were constructed and compared by using Bayesian model reduction scheme from the full design matrix. The model performance was evaluated with respect to free energy (i.e., approximation to log model evidence). In this study, four sessions from 30 individuals (in total 120 sessions) were evaluated. For each model (out of 32 models) for every session of each individual, we counted the frequency of the winning model. We then also performed Bayesian model comparison to assess the evidence for each model, which is accumulated by simply summing the free energy over subjects. We mostly found that the models that contained all the components at the second level performed better than any other combinations of temporal bases, except for fPCA. In the fPCA, the components up to 5th regressor (four dynamic regressors) were selected as a better model than any other combinations.

**Fig. 10:**
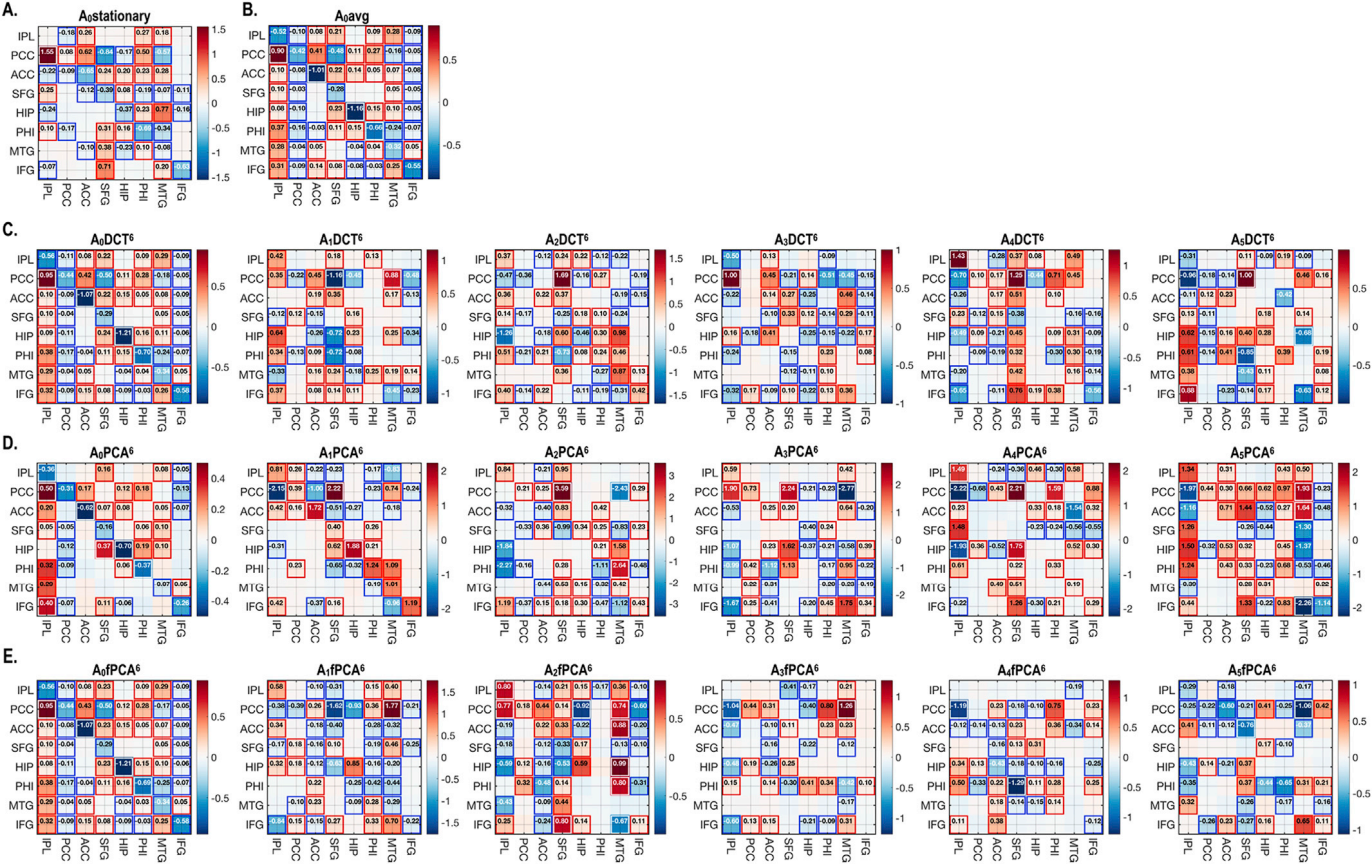
Group level effective connectivity of the default mode network for stationary and dynamic models. The intrinsic effective connectivity under stationarity assumption (*A*_0_stationary) (A) and the average model (*A*_0_avg) of all the windows in the session are averaged across sessions and participants using multilevel PEB. The group-level dynamic modes of effective connectivity are displayed according to regressor bases; DCT (C), PCA(D) and fPCA(E). Effect sizes that survived a criterion of 95% posterior confidence, or more, are shown in rectangles.

Fig. 10 shows group level effective connectivity of the DMN, comprising baseline and dynamic components averaged across sessions and individuals using multilevel PEBs. Depending on the choice of temporal basis set, the dynamic components of effective connectivity showed more or less variance. However, the baseline effective connectivity patterns (corresponding to the vector of ones in the second level design matrix) are very similar, particularly among *A*_0_avg, *A*_0_DCT^6^ and *A*_*0*_fPCA^6^. Compared to the adjacency matrix of the *A*_0_stationary, *A*_0_avg, *A*_0_DCT^6^ and *A*_*0*_fPCA^6^ showed a greater number of connections, with significant effective connectivity, particularly in the PCC, ACC and IFG. According to Fig. 10D, the baseline effective connectivity pattern in *A*_0_avg, *A*_0_DCT^6^ and *A*_*0*_fPCA^6^ could be decomposed into *A*_*0*_PCA^6^ and (negative) *A*_*1*_PCA^6^. In contrast to other basis sets, the second principal mode *A*_*1*_PCA^6^ was relatively stable across windows. The implicit conservation of *A*_*1*_PCA^6^ is apparent in Fig. 8C, which shows a relatively high between-session similarity for the second principal component (which was not found in the second components in DCT^6^ or fPCA^6^). Note that the simple average model (*A*_0_avg) identified a baseline effective connectivity that was conserved across sessions, as shown in Fig. 8B.

**Fig. 11:**
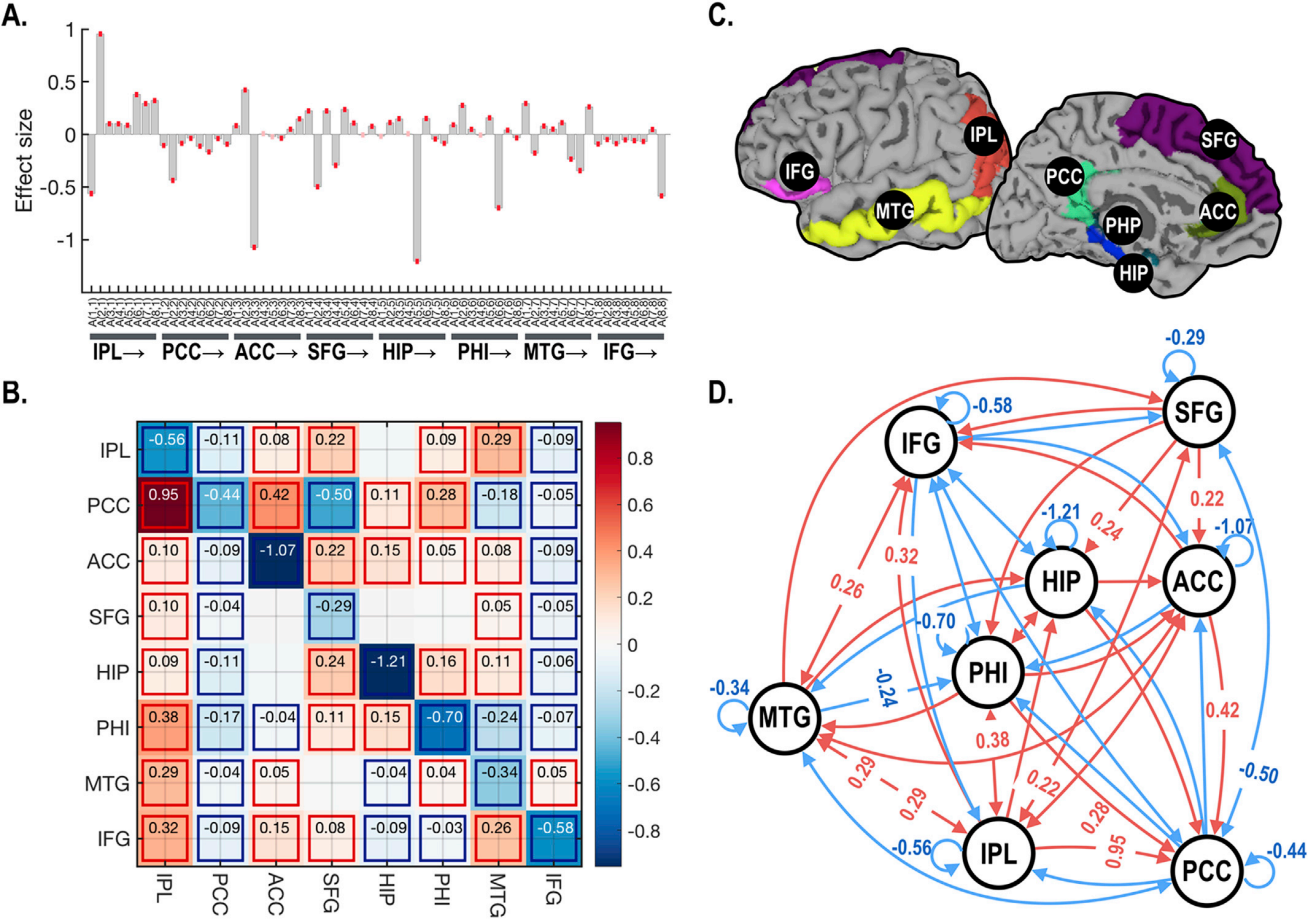
Group level baseline effective connectivity *A*_0_ (under the DCT model) of the default mode network. (A) Posterior expectation of parameters with confidence intervals and (B) its adjacency matrix of default mode brain regions (C). Effect sizes that survived a criterion of 95% posterior confidence, or more, are shown in rectangles. (D) Graphical illustration of the group level baseline effective connectivity. Red arrows indicate excitatory connection, whereas blue arrows show inhibitory connections. Effect sizes over 0.2 Hz are denoted in the graph. IPL: the inferior parietal lobe, PCC: posterior cingulate cortex, ACC: rostral anterior cingulate gyrus, SFG: superior frontal gyrus, HIP: hippocampus, PHP: parahippocampal gyrus, MTG: middle temporal gyrus and IFG: pars orbitalis of the inferior frontal lobe in the left hemisphere. Arrow after a region indicates directed connectivity from the region.

Fig. 11 summarizes the baseline intrinsic effective connectivity in the DCT model. In this baseline effective connectivity (Fig. 11), the posterior cingulate cortex and inferior frontal gyrus showed an inhibitory influence on the remaining DMN regions, while the inferior parietal lobe exerts positive influences on most brain regions within this network. A strong excitatory (positive) influence was estimated from the anterior to posterior cingulate cortex.

In the current study, model inversion took about an hour (with and without empirical priors using PEB) spDCMs for 11 windows on an Intel Xeon E5-2640 2.6 GHz.

### Discussion

In this paper, we have introduced a simple yet efficient method to characterize dynamic effective connectivity using parametric empirical Bayes (PEB) to model fluctuations in coupling over time. Using the proposed method (with DCM for resting state fMRI), we attempted to identify baseline and dynamic changes in the intrinsic effective connectivity of the default mode network. Crucially, the evidence for models that incorporate fluctuations in effective connectivity over time far exceeded those models that did not. Perhaps the most compelling evidence for the importance of dynamic effective connectivity is the remarkable disclosure of consistent patterns of baseline connectivity – over sessions – when, and only when dynamic effective connectivity components are modeled explicitly.

Recently, connectomics has devoted an increasing amount of attention to the dynamic nature of functional connectivity in resting-state fMRI (Allen et al., 2014, Calhoun et al., 2014, Chang and Glover, 2010, Cribben et al., 2012, Handwerker et al., 2012, Monti et al., 2014). These dynamics in functional connectivity have been extensively explored in terms of inter-regional temporal synchrony among endogenous BOLD fluctuations in distributed brain regions at rest. However, temporal synchrony can arise from a stimulus-locked common input or stimulus-induced oscillations through polysynaptic connections (Gerstein and Perkel, 1969), and therefore does not provide information about the casual influences among neural populations. To overcome this limitation, we used DCM (in particular, spectral DCM for rsfMRI), to account for both directed neuronal coupling and the subsequent mapping to haemodynamic responses, in the modelling of rsfMRI (Friston et al., 2014).

To estimate dynamic effective connectivity, we conducted hierarchical modelling of dynamic changes in effective connectivity within an individual. This method is an extension of the PEB framework to longitudinal modelling of DCMs (Friston et al., 2015, Friston et al., 2016); taking advantage of iterative Bayesian optimization procedures; i.e. empirical priors from the second (session) level iteratively optimize posterior densities over parameters at the first (window) level. This iterative approach finesses the local minima problem inherent in the inversion of nonlinear and ill-posed models; thus, it provides more robust and efficient estimates of within and between-window effects (Friston et al., 2015, Litvak et al., 2015).

The dynamic effective connectivity was then modeled using a between-window design matrix that comprised DCT or PCA basis functions of time. This hierarchical approach is conceptually similar to modelling described by Papadopoulou et al. (2017), where the spatiotemporal dynamics of seizure EEG activity were modeled using PEB of DCM with tonic and monotonic changes as temporal basis functions at the second level. However, in contrast to Papadopoulou et al. (2017), we did not use any external inputs or change markers (such as seizure makers) in the estimation of intrinsic connectivity dynamics.

In this study, we initially estimated the fully connected model (where each region in the DMN was connected to all the other regions) at the first level and used the full set of temporal regressors at the second level. We then compared models using Bayesian model reduction at the second level by constructing 32 models from the combination of 6 temporal bases of DCT, PCA or fPCA. Note that we could perform the model reduction both at the first and second level to infer the optimal neuronal architecture that best explains both the first and the second level (Friston et al., 2016). We can further derive Bayesian model averages for intrinsic effective connectivity estimation, averaging over all possible combinations of models at both the first and second level. This reflects the advantage of using PEB-based dynamic connectivity analysis to assimilate information (and uncertainty) that multiple hierarchical levels.

To generalize the dynamics of intrinsic effective connectivity at the group level, we used multi-level PEB analyses to characterize between-window (within session), between-session (within subject), and between-subject group effects. The PEB scheme allows for parametric random effects on connection strengths, between windows, sessions or subjects, in contrast to random effects on models *per se* (Stephan et al., 2010). This is clearly advantageous in the quantitative analysis of effective connectivity changes – and in terms of longitudinal mixed-effects. Again, this multilevel approach can be better than a fixed effect approach (i.e., simply concatenating data from different subjects to invert a group-level model), as conducted in Papadopoulou et al. (2017).

We have established the presence of dynamics in intrinsic effective connectivity by decomposing the intrinsic effective connectivity into baseline and dynamic components. In this study, we modeled the dynamic component with a linear combination of orthogonal temporal functions. As expected, we observed that the baseline effective connectivity was stable across different sessions, while the dynamic components fluctuated within a session varied across sessions.

spDCM for rsfMRI estimates effective connectivity from the cross-spectra of a given time-series (summarized using a multi-variate autoregressive (MAR) process). MAR for multichannel time series are generally valid for stationary conditions, which may be violated for longer time series. As described above, the dynamic nature of resting state connectivity, particularly for longer time series, as those found in HCP database, may not be sufficiently modeled by estimating the cross-spectra of the whole-time series. Instead of modelling the whole time-series under a stationary assumption, we estimated the cross-spectra (using MAR) of segmented time-series.

The simplest way to deal with multiple sampled cross spectra (from each window) is to simply average them in a PEB analysis. This approach regards the non-stationarity of the time series within a window as a (Gaussian) noise process relative to the stationary component (the stable part of connectivity matrix A). However, this simple model gives information only about the baseline *A*_0_ connectivity and does not account for the dynamic nature of effective connectivity within each window. Hence, we introduce temporal basis functions that model dynamic components of intrinsic effective connectivity using DCT, PCA or fPCA to account for the putative non-stationary component of the time series.

The current study demonstrates that changes in effective connectivity induced a non-stationarity in measured times series (and dynamic functional connectivity), which can be accounted for when we model those changes over short time scales (using windowing). Bayesian model comparison shows that models of between window effects – with dynamic regressors – had higher log evidence (free energy) than models without. This indicates that there are non-stationary components that cannot be simply modeled as random variations from the baseline connectivity (i.e., model 1 or model 2 (regressors up to 5 or 6th components) compared to the model 32 (only the first component) in Fig. 9).

Consistency across sessions in the baseline effective connectivity *A*_0_ also supports the dynamic nature of intrinsic effective connectivity. Compared to the stationary model of the entire time series, *A*_0_ in all non-stationary models showed significantly higher consistency across sessions. The second or higher order components of DCT and fPCA show high variation across sessions and the degrees of the inter-session variability are similar across components except for the baseline component. It is of note that PCA based analysis of dynamic effective connectivity showed a relatively high cross-session similarity for the second (the first dynamic regressor) principal eigenvariate. This may seem counterintuitive; however, the eigenvariates were subject-specific; suggesting that subject specific changes in effective connectivity are conserved over sessions. The interesting thing here is that the same connections seem to be involved in these systematic time effects.

The default mode network (DMN), an intrinsic brain network, deactivates when the brain engages in an attention demanding task (Raichle et al., 2001). The DMN is generally composed of the posterior cingulate cortex or precuneus, anterior cingulate cortex or medial frontal cortex, hippocampus, and the inferior parietal lobe. There are several studies that have explored intrinsic effective connectivity of DMN using DCM, either using stochastic DCM (Li et al., 2012) or spectral DCM (Di and Biswal, 2014, Razi et al., 2015, Sharaev et al., 2016b, Ushakov et al., 2016). In the current study, we conducted spDCM for a larger and extended DMN (that includes 8 nodes in a hemisphere). We found a relatively weak (<0.1 Hz) but statistically significant inhibitory influence from the PCC to other DMN regions in the baseline connectivity of the dynamic model. This is partly consistent with previous findings that showed anti-correlation between PCC and other brain regions. For example, Razi et al. (2015) and Sharaev et al. (2016a) compared models comprising four nodes (PCC, left and right IPL and medial prefrontal cortex(mPFC)) with spectral DCM and found that IPL received inhibitory coupling from PCC, while mPFC has mainly excitatory coupling. Di and Biswal (2014) showed there was no effective connection from PCC to other areas; however, the authors did not include neural fluctuations in the neural state equation and only modeled endogenous activity in low-frequency fluctuations. One might conclude that the intrinsic effective connectivity of the DMN still requires further study.

To characterize dynamic effective connectivity, we used a sliding-window approach, which is generally used in studies of dynamic (functional) connectivity (Allen et al., 2014, Calhoun et al., 2014, Chang and Glover, 2010, Cribben et al., 2012, Handwerker et al., 2012, Monti et al., 2014, Wang et al., 2016). In the sliding window approach, proper selection of the window size remains a challenge to optimally capture dynamics (Hindriks et al., 2016, Shakil et al., 2016). Here, we chose a window size of 200; according to the previous evaluation by Razi et al. (2015), which affords relatively stable estimation of intrinsic effective connectivity using resting state fMRI. However, optimal window selection remains a challenge that should be further explored in future studies; perhaps by varying the size and form of windowing function (for e.g. using a Gaussian window instead of the rectangular window used in this work). Such aspects of hierarchical modelling can then be optimized using Bayesian model selection. With regard to the window size, the optimal (or adaptive) choice of the order of MAR (we used fourth order MAR process) for given window size and the choice of optimal temporal basis functions remains outstanding goals. Furthermore, it may be possible to apply the current method over the segments identified by the change point detection method (Jeong et al., 2016).

In addition to endogenous fluctuations, recent studies imply that motion artifacts may also have an impact on the short-term cross-correlation structure of rsfMRI (Power et al., 2015). Discontinuous changes in the connectivity matrices around abrupt head motion events could occur either by the remaining (micro)motion artifacts or by true neurobiological changes in brain state that cause, or are caused by head movements (Yan et al., 2013, Zeng et al., 2014). These artefactual covariance changes could be reduced by carefully regressing out head movements and global signals. This motion artifact may lead to some spurious effects on the estimation of cross spectra using MAR. However, by subdividing the time series in smaller epochs, the new approach could ameliorate the effects of motion artifacts by Bayesian averaging of effective connectivity hence suppressing their effect. Crucially, motion artifacts affect all brain regions at the same time and therefore cannot be explained by the state space model used in dynamic causal modelling. In other words, effective connectivity can only explain changes in the rate of *change of signal* in one area as a function of signal in another; thereby rendering DCM relatively immune to these sort of artifacts (that are absorbed into global fluctuations).

The current study offers a proof-of-concept (and provides a face validation) for a hierarchical Bayesian characterization of dynamic effective connectivity. There are several outstanding issues that will be addressed in future studies; for example, the optimal number of temporal basis functions, the optimal length, number, and overlap of the sliding windows – and the selection of alternative second level prior distributions. In particular, the number of temporal basis functions is an important issue: here, we limited the number of temporal basis functions (in the second level model) to less than six, to focus on slow fluctuations in effective connectivity and suppress model complexity. This number was an empirical choice that represents a compromise between complete characterization (of effective connectivity changes) and the number of free parameters to be estimated. Beside this, many details remain to be resolved by further studies that could improve the reliability of the proposed method. In particular, we hope to demonstrate the predictive validity of the proposed method in clinical studies.

In summary, human brain networks at rest show dynamic functional connectivity that is induced by dynamic effective connectivity, which can be modeled efficiently using dynamic causal modelling and hierarchical Bayesian inference.

## References

[bib1] Allen E.A., Damaraju E., Plis S.M., Erhardt E.B., Eichele T., Calhoun V.D. (2014). Tracking whole-brain connectivity dynamics in the resting state. Cereb. Cortex.

[bib2] Biswal B., Yetkin F.Z., Haughton V.M., Hyde J.S. (1995). Functional connectivity in the motor cortex of resting human brain using echo-planar MRI. Magn. Reson Med..

[bib3] Bullmore E., Long C., Suckling J., Fadili J., Calvert G., Zelaya F., Carpenter T.A., Brammer M. (2001). Colored noise and computational inference in neurophysiological (fMRI) time series analysis: resampling methods in time and wavelet domains. Hum. Brain Mapp..

[bib4] Calhoun V.D., Miller R., Pearlson G., Adali T. (2014). The chronnectome: time-varying connectivity networks as the next frontier in fMRI data discovery. Neuron.

[bib5] Chang C., Glover G.H. (2010). Time-frequency dynamics of resting-state brain connectivity measured with fMRI. Neuroimage.

[bib6] Cooray G.K., Sengupta B., Douglas P.K., Friston K. (2016). Dynamic causal modelling of electrographic seizure activity using Bayesian belief updating. Neuroimage.

[bib7] Cribben I., Haraldsdottir R., Atlas L.Y., Wager T.D., Lindquist M.A. (2012). Dynamic connectivity regression: determining state-related changes in brain connectivity. Neuroimage.

[bib8] Desikan R.S., Segonne F., Fischl B., Quinn B.T., Dickerson B.C., Blacker D., Buckner R.L., Dale A.M., Maguire R.P., Hyman B.T., Albert M.S., Killiany R.J. (2006). An automated labeling system for subdividing the human cerebral cortex on MRI scans into gyral based regions of interest. Neuroimage.

[bib9] Di X., Biswal B.B. (2014). Identifying the default mode network structure using dynamic causal modeling on resting-state functional magnetic resonance imaging. Neuroimage.

[bib10] Friston K., Mattout J., Trujillo-Barreto N., Ashburner J., Penny W. (2007). Variational free energy and the Laplace approximation. Neuroimage.

[bib11] Friston K., Zeidman P., Litvak V. (2015). Empirical Bayes for DCM: a group inversion scheme. Front. Syst. Neurosci..

[bib12] Friston K.J. (2011). Functional and effective connectivity: a review. Brain Connect..

[bib13] Friston K.J., Harrison L., Penny W. (2003). Dynamic causal modelling. Neuroimage.

[bib14] Friston K.J., Kahan J., Biswal B., Razi A. (2014). A DCM for resting state fMRI. Neuroimage.

[bib15] Friston K.J., Litvak V., Oswal A., Razi A., Stephan K.E., van Wijk B.C., Ziegler G., Zeidman P. (2016). Bayesian model reduction and empirical Bayes for group (DCM) studies. Neuroimage.

[bib16] Gerstein G.L., Perkel D.H. (1969). Simultaneously recorded trains of action potentials: analysis and functional interpretation. Science.

[bib17] Glasser M.F., Sotiropoulos S.N., Wilson J.A., Coalson T.S., Fischl B., Andersson J.L., Xu J., Jbabdi S., Webster M., Polimeni J.R., Van Essen D.C., Jenkinson M., Consortium, W.U.-M.H (2013). The minimal preprocessing pipelines for the Human Connectome Project. Neuroimage.

[bib18] Greicius M.D., Krasnow B., Reiss A.L., Menon V. (2003). Functional connectivity in the resting brain: a network analysis of the default mode hypothesis. Proc. Natl. Acad. Sci. U. S. A..

[bib19] Handwerker D.A., Roopchansingh V., Gonzalez-Castillo J., Bandettini P.A. (2012). Periodic changes in fMRI connectivity. Neuroimage.

[bib20] He B.J., Zempel J.M., Snyder A.Z., Raichle M.E. (2010). The temporal structures and functional significance of scale-free brain activity. Neuron.

[bib21] Hindriks R., Adhikari M.H., Murayama Y., Ganzetti M., Mantini D., Logothetis N.K., Deco G. (2016). Can sliding-window correlations reveal dynamic functional connectivity in resting-state fMRI?. Neuroimage.

[bib22] Hutchison R.M., Womelsdorf T., Gati J.S., Everling S., Menon R.S. (2013). Resting-state networks show dynamic functional connectivity in awake humans and anesthetized macaques. Hum. Brain Mapp..

[bib23] Jeong S.O., Pae C., Park H.J. (2016). Connectivity-based change point detection for large-size functional networks. Neuroimage.

[bib24] Li B., Wang X., Yao S., Hu D., Friston K. (2012). Task-dependent modulation of effective connectivity within the default mode network. Front. Psychol..

[bib25] Li G., Shen H.P., Huang J.H.Z. (2016). Supervised sparse and functional principal component analysis. J. Comput. Graph. Statistics.

[bib26] Litvak V., Garrido M., Zeidman P., Friston K. (2015). Empirical Bayes for group (DCM) studies: a reproducibility study. Front. Hum. Neurosci..

[bib27] Lowe M.J., Mock B.J., Sorenson J.A. (1998). Functional connectivity in single and multislice echoplanar imaging using resting-state fluctuations. NeuroImage.

[bib28] Maxim V., Sendur L., Fadili J., Suckling J., Gould R., Howard R., Bullmore E. (2005). Fractional Gaussian noise, functional MRI and Alzheimer's disease. Neuroimage.

[bib29] McGuire P.K., Paulesu E., Frackowiak R.S., Frith C.D. (1996). Brain activity during stimulus independent thought. Neuroreport.

[bib30] Monti R.P., Hellyer P., Sharp D., Leech R., Anagnostopoulos C., Montana G. (2014). Estimating time-varying brain connectivity networks from functional MRI time series. Neuroimage.

[bib31] Papadopoulou M., Cooray G., Rosch R., Moran R., Marinazzo D., Friston K. (2017). Dynamic causal modelling of seizure activity in a rat model. Neuroimage.

[bib32] Park H.J., Friston K. (2013). Structural and functional brain networks: from connections to cognition. Science.

[bib33] Power J.D., Schlaggar B.L., Petersen S.E. (2015). Recent progress and outstanding issues in motion correction in resting state fMRI. Neuroimage.

[bib34] Raichle M.E., MacLeod A.M., Snyder A.Z., Powers W.J., Gusnard D.A., Shulman G.L. (2001). A default mode of brain function. Proc. Natl. Acad. Sci. U. S. A..

[bib35] Raichle M.E., Snyder A.Z. (2007). A default mode of brain function: a brief history of an evolving idea. NeuroImage.

[bib36] Razi A., Friston K. (2016). The Connected Brain: causality, models, and intrinsic dynamics. IEEE Signal Process. Mag..

[bib37] Razi A., Kahan J., Rees G., Friston K.J. (2015). Construct validation of a DCM for resting state fMRI. Neuroimage.

[bib38] Shakil S., Lee C.H., Keilholz S.D. (2016). Evaluation of sliding window correlation performance for characterizing dynamic functional connectivity and brain states. Neuroimage.

[bib39] Sharaev M.G., Zavyalova V.V., Ushakov V.L., Kartashov S.I., Velichkovsky B.M. (2016). Effective connectivity within the default mode network: dynamic causal modeling of resting-state fMRI data. Front. Hum. Neurosci..

[bib40] Sharaev M.G., Zavyalova V.V., Ushakov V.L., Kartashov S.I., Velichkovsky B.M. (2016). Effective connectivity within the default mode network: dynamic causal modeling of resting-state fMRI data. Front. Hum. Neurosci..

[bib42] Stephan K.E., Penny W.D., Moran R.J., den Ouden H.E., Daunizeau J., Friston K.J. (2010). Ten simple rules for dynamic causal modeling. Neuroimage.

[bib43] Stephan K.E., Weiskopf N., Drysdale P.M., Robinson P.A., Friston K.J. (2007). Comparing hemodynamic models with DCM. Neuroimage.

[bib44] Ushakov V., Sharaev M.G., Kartashov S.I., Zavyalova V.V., Verkhlyutov V.M., Velichkovsky B.M. (2016). Dynamic causal modeling of hippocampal links within the human default mode network: lateralization and computational stability of effective connections. Front. Hum. Neurosci..

[bib45] Van Essen D.C., Ugurbil K., Auerbach E., Barch D., Behrens T.E., Bucholz R., Chang A., Chen L., Corbetta M., Curtiss S.W., Della Penna S., Feinberg D., Glasser M.F., Harel N., Heath A.C., Larson-Prior L., Marcus D., Michalareas G., Moeller S., Oostenveld R., Petersen S.E., Prior F., Schlaggar B.L., Smith S.M., Snyder A.Z., Xu J., Yacoub E., Consortium, W.U.-M.H (2012). The Human Connectome Project: a data acquisition perspective. Neuroimage.

[bib46] Wang C., Ong J.L., Patanaik A., Zhou J., Chee M.W. (2016). Spontaneous eyelid closures link vigilance fluctuation with fMRI dynamic connectivity states. Proc. Natl. Acad. Sci. U. S. A..

[bib47] Yan C.G., Cheung B., Kelly C., Colcombe S., Craddock R.C., Di Martino A., Li Q., Zuo X.N., Castellanos F.X., Milham M.P. (2013). A comprehensive assessment of regional variation in the impact of head micromovements on functional connectomics. Neuroimage.

[bib48] Yeo B.T., Krienen F.M., Sepulcre J., Sabuncu M.R., Lashkari D., Hollinshead M., Roffman J.L., Smoller J.W., Zollei L., Polimeni J.R., Fischl B., Liu H., Buckner R.L. (2011). The organization of the human cerebral cortex estimated by intrinsic functional connectivity. J. Neurophysiol..

[bib49] Zeng L.L., Wang D., Fox M.D., Sabuncu M., Hu D., Ge M., Buckner R.L., Liu H. (2014). Neurobiological basis of head motion in brain imaging. Proc. Natl. Acad. Sci. U. S. A..

